# Biological and Methotrexate Survival after Pregnancy in Patients With a Rheumatic Disease

**DOI:** 10.3389/fphar.2022.826034

**Published:** 2022-03-09

**Authors:** Helena Tahmasian, Hieronymus T. W. Smeele, Pascal H.P. de Jong, Radboud J. E. M. Dolhain, Elise van Mulligen

**Affiliations:** ^1^ Department of Rheumatology, Erasmus University Medical Center, Rotterdam, Netherlands; ^2^ Department of Rheumatology, Leiden University Medical Center, Leiden, Netherlands

**Keywords:** biological, rheumatic dieases, pregnancy, biological survival, DMARD (disease modifying anti-rheumatic drug)

## Abstract

**Objective:** Patients with a rheumatic disease who discontinue their disease-modifying anti-rheumatic drug (DMARD) due to pregnancy often wonder if treatment will be as effective after pregnancy. This study investigates the effect of a temporary discontinuation of DMARDs due to pregnancy on the effectiveness of the same DMARD postpartum in patients with a rheumatic disease.

**Methods:** Pregnant, rheumatic patients were derived from the Preconceptional Counseling in Active Rheumatoid Arthritis (PreCARA) cohort. DMARD-survival after pregnancy, for biological and methotrexate (MTX) therapy, was analyzed and compared to controls with stable DMARD-treatment from a retrospective cohort.

**Results:** In total, 234 patients were included, of whom 114 patients had stable biological or MTX treatment before their pregnancy. After pregnancy, 40 out of 56 (71%) patients restarted their biological, for MTX this was 49%. One year after restart, and censoring for a following pregnancy, 88.9% of patients were still using their biological, and 85% still used their MTX (*p* = 0.92). Compared to the matched controls the survival after pregnancy was significantly lower 1 year after restart for both biologicals (98.3%) and MTX (99.6%); *p* = 0.002 and *p* < 0.001 respectively; 3 years after restart this significant difference was no longer observed (*p* = 0.50 and *p* = 0.33, respectively).

**Conclusion:** Effective DMARD (biological or MTX) treatment before pregnancy that was discontinued due to pregnancy seems effective after pregnancy. Although DMARD-survival was higher in the control group 1 year after restart, the percentage of patients with effective treatment was still very good (>85%). In addition, this difference was no longer observed after 3 years.

## Introduction

Rheumatic diseases are multifactorial autoimmune disorders with an unknown etiology, primarily affecting the joints.(Smolen et al., 2016; Radu and Bungau, 2021). The treatment of rheumatic diseases has improved tremendously over the last decades, and now includes early initiation of disease-modifying anti-rheumatic drugs (DMARDs), a treat-to-target approach, and use of biologicals.(Smolen et al., 2016).

A special group of autoimmune rheumatic disease patients are formed by patients with a wish to conceive or who are pregnant. Besides hormonal and immunological changes due to pregnancy, treatment of the rheumatic disease becomes a challenge within this patients group due to non-pregnancy compatible DMARDs.(Pacini et al., 2020). Nowadays, tumor necrosis factor alpha (TNFα) inhibitors are a vital part of treatment during pregnancy, whereas other DMARDs such as methotrexate (MTX) are incompatible with pregnancy (de Jong and Dolhain, 2017; Smeele et al., 2021) Pregnant patients are therefore compelled to discontinue their treatment, and will often restart after giving birth.

Previous literature showed that biological survival decreases with the number of biologicals a patient has used.(van Mulligen et al., 2021). In addition, interruption of biological treatment is associated with a reduced clinical response (Thomas et al., 2015; Favalli et al., 2017; Rubin, 2019). In line with that observation, patients and treating rheumatologists often question whether DMARD-treatment after pregnancy will be as effective as before the pregnancy.

Currently, it is unknown whether a temporary discontinuation of DMARD-treatment due to pregnancy affects efficacy of the same DMARD after pregnancy. Previous studies have not yet provided a clear answer of efficacy of a restarted DMARD after pregnancy, while it is a frequently asked patient question during pregnancy counseling. Therefore, the aim of this study is to investigate the effect of a temporary discontinuation of effective DMARD-treatment before pregnancy on the survival of this DMARD after pregnancy in patients with a rheumatic disease.

## Methods

### Patients

#### Pregnant Patient Group

For the current study, we used pregnant patients from the Preconceptional Counseling in Active Rheumatoid Arthritis (PreCARA) cohort. The study protocol of PreCARA-cohort has been described extensively previously.(Smeele et al., 2021). In short, it is an ongoing, long-term, prospective cohort from a tertiary referral center on inflammatory rheumatic diseases and pregnancy. The PreCARA-cohort investigated the feasibility of a modern treat-to-target treatment approach, including the use of TNF inhibitors, aiming for remission or low disease activity (LDA) in rheumatic disease patients with a wish to conceive, or who are pregnant. Within the treatment protocol the pregnancy, previous response on treatment, adverse events, and patient preference were taken into account. First, sulfasalazine or hydroxychloroquine was started, followed by addition of prednisone or a TNF inhibitor, preferably certolizumab pegol. Patients could get pregnant using the TNF inhibitor on which they enrolled, though TNF inhibitors were stopped during pregnancy at the gestational age as advised by the EULAR, (Gotestam Skorpen et al., 2016; Smeele et al., 2021). Adalimumab and infliximab were preferably stopped at 20 weeks, and etanercept at week 30–32 of pregnancy. Certolizumab was used throughout pregnancy until week 38, to prevent maternal complication during delivery.(Gotestam Skorpen et al., 2016). After stopping, a switch to certolizumab or prednisone was considered. For the current analysis, PreCARA-patients with stable, effective biological or MTX treatment before pregnancy who discontinued due to a wish to conceive or pregnancy, and restarted their treatment after pregnancy, were selected. Effective treatment was defined as a continuous treatment duration for at least 1 year.

#### Control Group

For our control group, a selection was made from a retrospective biological cohort, which was described earlier.(van Mulligen et al., 2021). We selected controls based on (female) gender, and age by excluding males and patients older than 40. In addition, patients who gave birth, and patients who did not have a continuous biological or MTX treatment for the duration of at least 1 year were excluded. Subsequently, our control group consisted of non-pregnant women with a rheumatic disease who had stable biological or MTX treatment of at least 1 year.

### Data Collection

Patients were enrolled from August 2011 onwards. Data collected concerning DMARD use were frequencies, start and stop dates, and reasons for discontinuation. Furthermore, risk factors for DMARD discontinuation were collected at baseline. In case non-adherence was reported, or start and/or stop dates of DMARDs were missing, patients were excluded from the analyses. Reasons for discontinuation were evaluated and classified into inefficacy; adverse events (AEs); remission; pregnancy; and patient preference.

In case patients had been referred back to the rheumatologist that treated them before pregnancy, in accordance with the PreCARA-protocol 6 months after delivery, we requested additional information from their treating physician on DMARD use in order to further extend the follow-up period.

### Data Analysis and Statistics

DMARD survival after pregnancy, for biological survival and MTX survival were compared using Kaplan-Meier survival curves, and analyzed with Logrank tests. To take a following pregnancy into account, censoring was performed on patients who discontinued DMARD treatment due to a following pregnancy. Subsequently, DMARD survival postpartum was compared to DMARD survival after 1 year of stable treatment in the control group. The percentage of DMARD survival 1 year and 3 years after restart were compared between the investigation and control group. Lastly, these analyses were stratified for reason for discontinuation.

All data were analyzed using STATA v16 (StataCorp-LP). *p* values ≤0.05 were considered statistically significant.

### Patient Participation and Ethics

The PreCARA study is conducted in a tertiary referral center, the Erasmus MC. Within the rheumatology department, patients are actively consulted for input on research questions. For the current study the pregnancy patient panel was consulted. Patients in this panel discussed the current research questions and stated that effectiveness of treatment is highly important, especially during and after pregnancy.

All patients of the PreCARA-cohort gave written informed consent, for the retrospective biological study no informed consent was needed. The PreCARA and the retrospective biological cohort were both approved by the Erasmus MC ethics review board (MEC-2011-032 and MEC-2019-0573, respectively), and were executed in compliance with the Helsinki Declaration.

## Results

### Patients

**FIGURE 1:**
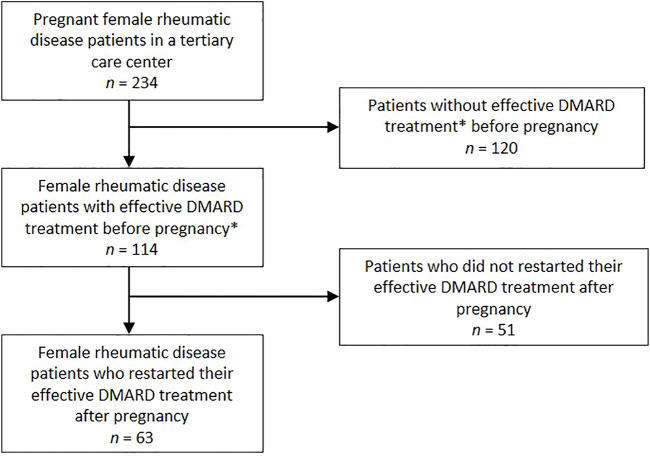
Flowchart of pregnant patient selection from the PreCARA cohort. * Effective treatment was defined as continuous treatment for at least 1 year.

The selection of patients is shown in Figure 1. The characteristics of the rheumatic disease pregnant patient population and the control group are shown in Table 1. Of the female rheumatic disease patients who got pregnant, 32 patients (51%) were diagnosed with rheumatoid arthritis (RA), the other diagnoses were spondyloarthropathy (13%), psoriatic arthritis (21%), and juvenile idiopathic arthritis (16%). 220 (56%) patients in the control group were diagnosed with RA. Mean age (SD) at diagnosis differed significantly between both groups (*p* < 0.001), respectively 22.5 (8) years and 33.9 (17) years for the investigation group and the control group.

**TABLE 1:** Characteristics of rheumatic disease patient population and control group in a university hospital

—	Female rheumatic disease patients who got pregnant, *n* = 63	Female rheumatic disease patients control group, *n* = 395
Diagnosis		
Rheumatoid arthritis, n (%)	32 (51)	220 (56)
- ACPA positive, n (%)	25 (78)	162 (74)
- RF positive, n (%)	27 (84)	156 (71)
- Erosive disease, n (%)	12 (38)	96 (44)
Spondyloarthropathy	8 (13)	46 (12)
- HLAB27 positive, n (%)	2 (25)	28 (61)
- Erosive disease, n (%)	2 (25)	1 (2.2)
Psoriatic arthritis, n (%)	13 (21)	64 (16)
- Erosive disease, n (%)	1 (8)	12 (19)
Juvenile idiopathic arthritis, n (%)	10 (16)	54 (14)
- Erosive disease, n (%)	3 (30)	7 (13)
Undifferentiated arthritis, n (%)	0	11 (2.8)
Demographic		
Age at diagnosis, mean (SD)^*^	22.5 (8)	33.9 (17)
Follow-up (years) from first biological, median (IQR)	8.9 (5-11)	6.3 (3-10)
BMI, mean (SD)	25.0 (5)	26.5 (6.4)
Medication		
Time to first biological (years), mean (SD)	4.3 (6)	5.2 (0.4)
Any use of anti-TNFα		
- Adalimumab, n (%)	30 (52)	153 (39)
- Certolizumab, n (%)	32 (51)	79 (20)
- Etanercept, n (%)	41 (65)	145 (37)
- Golimumab, n (%)	3 (5)	26 (6.6)
- Infliximab, n (%)	13 (21)	43 (11)
Any use of non anti-TNFα biological		
- IL6i, n (%)	9 (14)	21 (5.3)
- IL17i, n (%)	3 (5)	12 (3.0)
- JAKi, n (%)	1 (2)	13 (3.3)
- oMOA, n (%)	6 (10)	25 (6.3)
Any use of conventional DMARDs		
- MTX	54 (86)	244 (62)
- MTX + SASP and/or HCQ, n (%)	48 (76)	229 (58)
- Other csDMARDs, n (%)	37 (59)	95 (24)
Any use of corticosteroids	33 (52)	55 (14)

*p < 0.001.

ACPA, anti-citrullinated protein antibody; BMI, body mass index; csDMARD, conventional synthetic disease-modifying anti-rheumatic drug; HCQ, hydroxychloroquine, IL6i Interleukin-6, inhibitor, IL17i Interleukin-17, inhibitor; JAKi Janus Kinase inhibitor; MTX, methotrexate; oMOA, other mechanism of action; SASP, sulfasalazine; RF, rheumatoid factor; SD, standard deviation.

### Restart of Previous Effective MTX or Biological Therapy After Pregnancy

In total, 114 patients had effective DMARD (biological or MTX) treatment before their pregnancy according to our definition (Figure 1). Of these 114 patients, 41 used MTX only, 56 used biological monotherapy, and 17 used both MTX and a biological. After pregnancy, 63 of these 114 patients (56%) restarted the exact same treatment (Table 1). Of the 56 patients who had effective biological monotherapy before pregnancy, 40 patients (71%) restarted their treatment after pregnancy. The percentage of patients who restarted their effective MTX treatment after pregnancy was 49%.

### MTX and Biological Survival After Pregnancy

**FIGURE 2:**
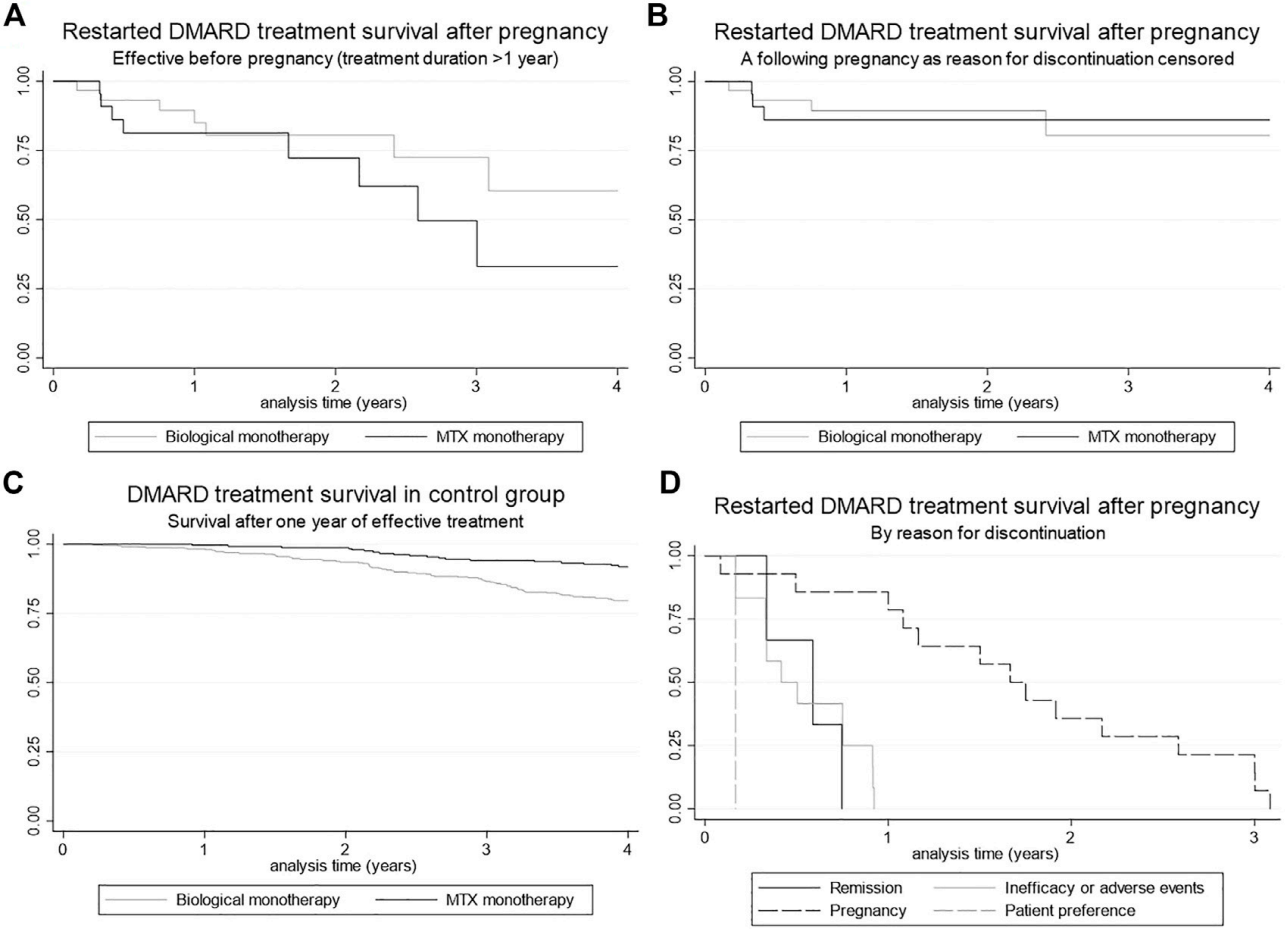
Kaplan Meier curves for DMARD survival **(A)** Restarted DMARD treatment (biological and MTX) survival after pregnancy, **(B)** Restarted DMARD treatment (biological and MTX) survival after pregnancy, censored for pregnancy as reason for discontinuation, **(C)** DMARD treatment (biological and MTX) survival within the control group after 1 year of continuous treatment, **(D)** Restarted DMARD treatment survival after pregnancy, stratified for reason of discontinuation.

One year after restart of their biological, 88.9% of the patients still used this biological. 80.5% of the patients who restarted MTX were still using it 1 year after restart (Figure 2A). When we censored for a following pregnancy, percentages were 88.9 and 85.0%, respectively (Figure 2B). After 3 years, survival of the biological was still 80%, and for MTX 85%. Overall, there was no significant difference in survival between the biological and MTX survival group (*p* = 0.92, Figure 2B). To evaluate differences in standards of care which evolve over time, analyses were repeated for a subgroup with most recent data (from 2017 onwards) in comparison with a subgroup with less recent data (before 2017) (Supplementary Figure S1). No significant difference were found in survival in comparison to the main analysis.

Biological or MTX survival after 1 year of continuous treatment (t = 0) in the control group is shown in Figure 2C. After 1 year, 98.3% of the patients in the control group (patients that used DMARD treatment for 1 year continuously) still used this biological. This percentage was 99.6% for MTX. After 3 years these percentages were 86.6 and 94.1%, respectively.

Compared to the investigation group, MTX and biological survival was significantly higher in the control group for both biologicals and MTX (*p* = 0.002 and *p* < 0.001, respectively). Three years after restart no significant difference was found between the two groups for both biologicals and MTX (*p* = 0.50 and *p* = 0.33, respectively).

### Reasons for Discontinuation of DMARD Therapy

The reasons for discontinuation of DMARD therapy in the pregnant patient group are shown in Figure 2D. The main reason for discontinuation was a following pregnancy (50%). For the control group, the main reasons for discontinuation of biological monotherapy were inefficacy (57%), remission (19%) and adverse events (14%). Discontinuation reasons for MTX monotherapy were adverse events (56%), remission (25%) and inefficacy (8%).

## Discussion

A reduced clinical response to biological treatment after an interruption in treatment has been observed in previous literature (Rubin, 2019). We investigated the influence of a temporarily interruption in biological or MTX treatment due to pregnancy in patients with a rheumatic disease.

Although DMARD-survival was slightly higher in the control group, >85% of pregnant patients continued DMARD therapy for >12 months, when this reintroduced after delivery. This indicates that the medication was still very effective, despite being interrupted temporarily due to pregnancy. Also, findings were in line with previous research on DMARD-effectiveness outside pregnancy.(7)

In the MTX group, a lower percentage of restart after pregnancy was observed compared to the biological group (49% compared to 71%). This difference could probably be explained by incompatibility of MTX with breastfeeding, and therefore resulting in a lower percentage of MTX restart after pregnancy.(de Jong and Dolhain, 2017). In addition, MTX is associated with adverse events, especially nausea, and might therefore be less appealing for patients to restart.(Wang et al., 2018).

Literature shows that a temporary discontinuation of DMARDs can be harmful, especially in case of biological DMARDs.(Rubin, 2019). Formation of antidrug antibodies after a temporary discontinuation of infliximab and adalimumab are regularly found in patients with inflammatory bowel disease (Rubin, 2019). These antidrug antibodies could lead to inefficacy of biologics and/or adverse events and might therefore cause biological treatment failure.(Thomas et al., 2015). We observed that the temporarily discontinuation of DMARDs during pregnancy may be associated with a slightly reduced clinical response of the biological DMARDs or MTX within the first year after restart. However, the biological and MTX treatment after pregnancy still seems effective in over 85% of patients. This is reassuring, and in line with our recently published results concerning modern treat-to-target treatment strategy within pregnant patients. (Smeele et al., 2021).

Until recently, it was assumed that pregnant patients with RA reach a state of remission during pregnancy independent of treatment due to natural immunosuppression. However, the opposite was shown; more than half of pregnant patients still has active disease during pregnancy.(de Man et al., 2008). Though, due to improved care by applying a treat-to-target strategy, 90% of pregnant patients can have low disease activity during pregnancy and postpartum. (Smeele et al., 2021).

Our study has several strengths. It is the first study to investigate survival of the most commonly used biologicals and MTX after pregnancy. Moreover, it provided an answer to a frequent patient question during pregnancy counseling. Furthermore, our study contains a large group of pregnant patients and controls, and has a long follow-up time.

Some limitations of our study need to be addressed as well. Firstly, all patients were recruited in a tertiary care center with tight clinical monitoring and therefore our results can probably not be extrapolated to a non-tertiary setting.(Smeele et al., 2021). Furthermore, the number of patients using biologicals with another mode of action than TNF-inhibitors was small, therefore we were unable to investigate the survival of this group of biologics separately. Lastly, even though best efforts were made to make the investigation group and control group comparable, mean age at diagnosis differed significantly between both groups.

Our study provides several new opportunities for future research. Future research should focus on confirming our findings in order to reach scientific consensus, ideally, this future research should be conducted in a prospective cohort study with a large sample size. Furthermore, future research could also look into combining DMARD survival with disease measures, such as the DAS and VAS pain to ensure that treatment effectiveness is similar before and after pregnancy. Also, since we were unable to draw firm conclusions about non-TNF-inhibitor biologicals, more patients using these biologicals should be studied to draw conclusions about their survival.

The results of our study could be implemented in clinical practice by being part of the preconception counseling of patients with a wish to conceive. Previous research has shown that well-informed patients and shared decision making improves the likelihood of good maternal and child outcomes (Ostensen, 2017). When patients do not receive proper pregnancy counseling, it could lead to misuse of DMARDs (Birru Talabi et al., 2021). Therefore, the use of DMARDs during and after pregnancy should always be discussed with patients with a wish to conceive. Patients questioning their DMARD effectiveness after pregnancy can, with the results of the current study in mind, be reassured.

In conclusion, effective DMARD (biological or MTX) treatment before pregnancy seems effective after pregnancy. Although DMARD-survival was significantly higher in the control group 1 year after restart, the percentage of patients with effective treatment was still very good (>85%). In addition, this difference was no longer observed after 3 years. During pregnancy counseling, our results could provide a basis for shared decision making between physicians and patients on DMARD use after pregnancy. Our study shows that during this shared decision making process physicians can reassure the patients about DMARD-effectiveness after pregnancy.

## Significance and Innovation

• Patients with a rheumatic disease who discontinue their biological or MTX due to pregnancy often wonder if their treatment will be as effective after pregnancy. Previous literature has already described the forming of antidrug antibodies after an interruption in biological treatment, possibly affecting treatment efficacy. The influence of a temporary discontinuation due to pregnancy on the survival after pregnancy has not been investigated yet.
• Effective biological or MTX treatment before pregnancy that was discontinued due to pregnancy seems effective after pregnancy.

## Data Availability

The raw data supporting the conclusions of this article are available upon reasonable request by the authors.

## References

[B1] Birru Talabi M. Eudy A. M. Jayasundara M. Haroun T. Nowell W. B. Curtis J. R. (2021). Tough Choices: Exploring Medication Decision-Making during Pregnancy and Lactation Among Women with Inflammatory Arthritis. ACR Open Rheumatol. 3, 475–483. 10.1002/acr2.11240 34114738 PMC8281053

[B2] de Jong P. H. Dolhain R. J. (2017). Fertility, Pregnancy, and Lactation in Rheumatoid Arthritis. Rheum. Dis. Clin. North. Am. 43, 227–237. 10.1016/j.rdc.2016.12.004 28390565

[B3] de Man Y. A. Dolhain R. J. Van De Geijn F. E. Willemsen S. P. Hazes J. M. (2008). Disease Activity of Rheumatoid Arthritis during Pregnancy: Results from a Nationwide Prospective Study. Arthritis Rheum. 59, 1241–1248. 10.1002/art.24003 18759316

[B4] Favalli E. G. Raimondo M. G. Becciolini A. Crotti C. Biggioggero M. Caporali R. (2017). The Management of First-Line Biologic Therapy Failures in Rheumatoid Arthritis: Current Practice and Future Perspectives. Autoimmun. Rev. 16, 1185–1195. 10.1016/j.autrev.2017.10.002 29037899

[B5] Götestam Skorpen C. Hoeltzenbein M. Tincani A. Fischer-Betz R. Elefant E. Chambers C. (2016). The EULAR Points to Consider for Use of Antirheumatic Drugs before Pregnancy, and during Pregnancy and Lactation. Ann. Rheum. Dis. 75, 795–810. 10.1136/annrheumdis-2015-208840 26888948

[B6] Østensen M. (2017). Preconception Counseling. Rheum. Dis. Clin. North. Am. 43, 189–199. 10.1016/j.rdc.2016.12.003 28390562

[B7] Pacini G. Paolino S. Andreoli L. Tincani A. Gerosa M. Caporali R. (2020). Epigenetics, Pregnancy and Autoimmune Rheumatic Diseases. Autoimmun. Rev. 19, 102685. 10.1016/j.autrev.2020.102685 33115633

[B8] Radu A. F. Bungau S. G. (2021). Management of Rheumatoid Arthritis: An Overview. Cells 10, 2857. 10.3390/cells10112857 34831081 PMC8616326

[B9] Rubin D. T. (2019). Restarting Biologic Agents after a Drug Holiday. Gastroenterol. Hepatol. (N Y) 15, 612–615. 10.1186/s12992-019-0498-2 31802986 PMC6883732

[B10] Smeele H. T. Roder E. Wintjes H. M. Kranenburg-Van Koppen L. J. Hazes J. M. Dolhain R. J. (2021). Modern Treatment Approach Results in Low Disease Activity in 90% of Pregnant Rheumatoid Arthritis Patients: the PreCARA Study. Ann. Rheum. Dis. 80, 859–864. 10.1136/annrheumdis-2020-219547 33568387 PMC8237196

[B11] Smolen J. S. Aletaha D. Mcinnes I. B. (2016). Rheumatoid Arthritis. Lancet 388, 2023–2038. 10.1016/S0140-6736(16)30173-8 27156434

[B12] Thomas S. S. Borazan N. Barroso N. Duan L. Taroumian S. Kretzmann B. (2015). Comparative Immunogenicity of TNF Inhibitors: Impact on Clinical Efficacy and Tolerability in the Management of Autoimmune Diseases. A Systematic Review and Meta-Analysis. BioDrugs 29, 241–258. 10.1007/s40259-015-0134-5 26280210

[B13] van Mulligen E. Ahmed S. Weel A. E. A. M. Hazes J. M. W. Van Der Helm-Van Mil A. H. M. De Jong P. H. P. (2021). Factors that Influence Biological Survival in Rheumatoid Arthritis: Results of a Real-World Academic Cohort from the Netherlands. Clin. Rheumatol. 40, 2177–2183. 10.1007/s10067-020-05567-6 33415451 PMC8121743

[B14] Wang W. Zhou H. Liu L. (2018). Side Effects of Methotrexate Therapy for Rheumatoid Arthritis: A Systematic Review. Eur. J. Med. Chem. 158, 502–516. 10.1016/j.ejmech.2018.09.027 30243154

