# Supplementary figures and images for: Biological and Methotrexate Survival after Pregnancy in Patients With a Rheumatic Disease
Source: Front Pharmacol. 2022 Mar 9;13:826034. doi: 10.3389/fphar.2022.826034 (PMC8959570; doi:10.3389/fphar.2022.826034)

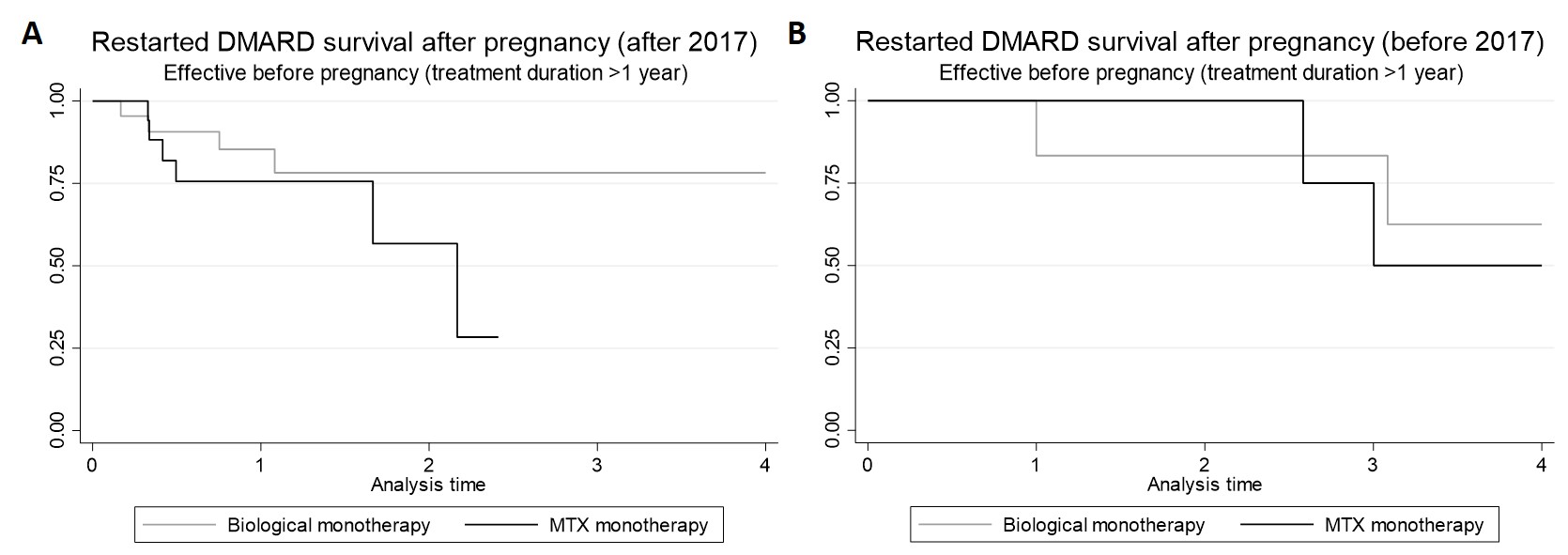

Supplement: Supplementary file 1 [file Image1.JPEG]
